# Patterns of Intensive Care Unit Mortality from Natural Causes at a Tertiary Care Centre in Western Nepal: An Observational Study

**DOI:** 10.31729/jnma.v63i292.9271

**Published:** 2025-12-31

**Authors:** Chetan Bohara, Arun Rimal, Parshal Bhandari, Laxmi Prasad Sapkota, Priska Bastola, Sakar Gyawali, Alok Atreya

**Affiliations:** 1 Department of Anaesthesia and Critical Care, Lumbini Medical College, Palpa, Nepal; 2 Lumbini Medical College, Palpa, Nepal; 3 Department of Community Medicine, Lumbini Medical College, Palpa, Nepal; 4 Department of Anesthesia, Maharajgunj Medical Campus, Kathmandu, Nepal; 5 Department of Forensic Medicine, Lumbini Medical College, Palpa, Nepal

**Keywords:** *comorbidity*, *critical care*, *diabetes mellitus*, *mortality*, *Nepal*

## Abstract

**Introduction:**

Intensive care units (ICUs) play a vital role in managing critically ill patients, but mortality rates remain high, especially in resource-limited settings like Nepal. This study aimed to describe the patterns and clinical characteristics of mortality cases in the ICU of a tertiary care hospital in western Nepal, with specific focus on sex-based differences and their associated comorbidities.

**Methods:**

A retrospective study was conducted at Lumbini Medical College Teaching Hospital, Nepal, over a one-year period (January 1 to December 31, 2024). This record-based study included mortality data of all ICU patients who died during their hospital stay and had complete medical records. Data on demographics, clinical parameters, comorbidities, laboratory findings, and need for mechanical ventilation were extracted using a structured proforma.

**Results:**

A total of 82 (21.93%) ICU mortality cases were included out of 374 ICU admissions, consisting of 45 (54.88%) male and 37 (45.12%) female. Median age was 63 (IQR: 46-73) years in males and 70 (IQR: 58-78) years in females. There was clustering of mortality, with 13 (15.85%) deaths occuring in September and 11 (13.41%) in November. The median age of diabetic patients was 72 (IQR: 66-77) years, while the median age of non-diabetic patients was 57.50 (IQR: 44 -73.50) years. The mean systolic blood pressure in diabetic patients was 117.79±22.64 mmHg, and in non-diabetic patients was 102.35±30.20 mmHg.

**Conclusions:**

The clustering of mortality was seen during a four-month period from August through November. Male patients constituted a majority of ICU deaths due to natural causes.

## INTRODUCTION

Intensive Care Units (ICUs) are essential for the management of critically ill patients but continue to face high mortality rates worldwide.^1,2^ The Global Burden of Disease (GBD) studies have demonstrated a major epidemiologic transition from infectious to non-communicable causes of death. Cardiovascular diseases, chronic respiratory disorders, stroke, diabetes, and malignancies have become the predominant contributors to global mortality.^3-6^ In South Asian countries, including Nepal, the burden of non-communicable diseases (NCDs) has risen rapidly in the past decade, contributing to an increased demand for intensive care services.

Despite the growing evidence from global databases, there remains limited national-level data on the pattern of ICU mortality in Nepal. Regional variations, socioeconomic barriers, and inconsistent ICU admission criteria further complicate the interpretation of existing studies.^7^ A detailed understanding of local mortality characteristics could therefore bridge this evidence gap and assist in policy formulation.

This study was therefore conducted to analyze mortality trends and contributing factors among ICU patients who died of natural cause at a tertiary care hospital in western Nepal. Secondary objectives included comparing clinical characteristics between male and female patients.

## METHODS

This is a retrospective, cross-sectional study conducted over a period of one year from 1 January to 31 December 2024. The study was conducted following ethical approval from the Institutional Review Committee of Lumbini Medical College (IRC-LMC:01/25). The study site was at Lumbini Medical College Teaching Hospital, Nepal, a tertiary care hospital in Lumbini province of western Nepal. The ICU at this center is a mixed medical-surgical unit with ten beds, each equipped with ventilatory support, multi-parameter monitors, infusion pumps, and basic laboratory and radiological support. The unit is staffed continuously by anesthesiologists, intensivists, residents, and trained critical care nurses, following a 24-hour rotation schedule. The ICU at this facility manages critically ill patients from both urban and rural areas, making it a representative setting for studying ICU mortality patterns in the region.

All ICU mortality during the study period were included in the study and cases with incomplete medical record and patients were transferred to another facility before the outcome (death or discharge) were excluded from the study. The unnatural cause of death requiring police notification and post-mortem examination to determine the cause of death were also excluded. The primary variables included demographic details, physiological parameters at admission, impaired consciousness (GCS), comorbid conditions, laboratory findings, and treatment interventions such as mechanical ventilation. Impaired consciousness was assessed by the Glasgow Coma Scale, with a minimal score of three and a maximum score of 15.^8^ A structured proforma was used for data collection. The data were systematically extracted from ICU records using the pre-designed proforma. Since this was a retrospective, record-based study, consent was not required.

The data were entered into a Microsoft Excel worksheet. The worksheet was then imported into the R software (version 4.4.1, R Foundation for Statistical Computing, Vienna, Austria software for data analysis. Continuous variables are reported as mean±standard deviation (SD) for normally distributed data or median (interquartile range, IQR) for skewed distributions. Categorical variables are presented as frequencies and percentages.

## RESULTS

Out of 374 ICU admissions, ICU mortality during the study period due to natural cause was 82 (21.93%). Out of total mortality 37 (45.12%) were female and 45 (54.88%) were male. The median age of female patients was 70.0 (IQR: 58.0-78.0) years, and male patients was 63.0 (IQR: 46.0-73.0) years. There was clustering of ICU mortality with 40 (48.78%) death occurring within a four-month period (August through November), (Table 1).

The length of ICU stay ranged from 1 day to 14 days, with an overall median duration of stay being 2.0 (IQR: 1.0-3.0) days.

The minimum GCS of 3 was recorded in 15 (18.29%) patients, and the maximum of 15 was recorded in 24 (29.27%), with a median score of 12.0 (IQR: 5.5-14.5). Among the vital signs, median systolic blood pressure in males was 110.0 (IQR: 90.0130.0) mmHg, while in females it was 95.0 (IQR: 80.0-110.0) mmHg. Similarly, median heart rate was 116.0 (IQR: 96.0-130.0) beats per minute (bpm) in males, while 86.0 (IQR: 76.0102.0) bpm in females. Median hemoglobin was 11.5 (IQR: 8.213.4) g/dL in females and 12.1 (IQR: 10.9-14.8) g/dL in males. Median potassium was 4.5 (IQR: 4.0-5.2) mmol/L in males and 3.9 (IQR: 3.6-4.5) mmol/L in females (Table 2).

**Table 1:** Time series analysis of mortality and deviation from median (n=82).

Month	Mortality n(%)	Deviation from median
January	3(3.66%)	-4
February	4(4.88%)	-3
March	7(8.54%)	0
April	7(8.54%)	0
May	4(4.88%)	-3
June	7(8.54%)	0
July	6(7.31%)	-1
August	8(9.76%)	+1
September	13(15.85%)	+6
October	8(9.76%)	+1
November	11(13.41%)	+4
December	4(4.88%)	-3

Median monthly mortality=7 cases; IQR=4-8 cases

**Table 2:** Baseline characteristics of male and female who had mortality in intensive care unit (n=82).

Characteristic	Female^*^ (n=37)	Male^*^ (n=45)
Age	70(58, 78)	63(46, 73)
Systolic BP	95(80, 110)	110(90, 130)
Diastolic BP	63(50, 80)	70(60, 90)
Heart rate	86(76, 102)	116(96, 130)
Respiratory rate	24(22, 26)	25(20, 31)
SpO2	92(84, 96)	91(82, 97)
GCS	12(6,14)	12(5, 15)
Hemoglobin	11.5(8.2, 13.4)	12.1(10.9, 14.8)
TLC	10,300(7,500, 14,900)	11,300(8,400, 14,000)
Platelet count	199,000(142,000, 253,000)	156,500(124,500, 222,000)
Urea	61.5(31.5, 85)	58(38, 75)
Creatinine	1.3(0.9, 2.2)	1.4(1, 1.9)
Sodium	141(138, 144)	140(138, 144)
Potassium	3.9(3.6, 4.5)	4.5(4, 5.2)

* Median(Q1,Q3); BP=Blood Pressure; GCS=Glasgow Coma Score; TLC: Total Lymphocyte Count

**Table 3:** Baseline characteristics of male and female who had mortality in intensive care unit (n=82).

Characteristic	Female (n=37)	Male (n=45)
Diabetes	7 (18.92%)	11 (24.44%)
Hypertension	10 (27.03%)	7 (15.56%)
Heart disease	4 (10.81%)	0 (0%)
Lung disease	11 (29.73%)	4 (8.89%)
Kidney disease	2 (5.41%)	1 (2.22%)
Liver disease	2 (5.41%)	1 (2.22%)
Cancer	0 (0%)	1 (2.22%)
Mechanical ventilation	22 (59.46%)	28 (62.22%)

## DISCUSSION

The finding of this study on ICU mortality patterns in a tertiary care centre in western Nepal, revealed an overall mortality rate of 21.93% (82/374 admissions). Sex-based differences were notable, with females showing significantly higher prevalence of heart disease and lung disease, alongside significantly lower systolic blood pressure and heart rate. Patients with diabetes were older and had higher systolic blood pressure than those without diabetes.

One of the most notable findings from this study is the significant temporal aggregation of ICU deaths with approximately half of the mortality in only a four-month period (between AugustNovember 2024). Monthly variability in the occurrence of ICU death, a non-uniform distribution, further supports that mortality is not evenly distributed over time but is influenced by time-specific factors. The clustering period in the present study has some overlap with monsoon in Nepal (June-August) and also with the winter season (November-January). Monsoon is linked to increased water-borne infections while cold and stus are more common during winter months. The detected clustering implies that the disease burden might be affected by the effects of weather-transition, indoor air pollution dynamics, and/or pattern of usage for healthcare facilities.^9^

These findings are in agreement with the global trend from the GBD studies, where NCDs now account for the majority of deaths worldwide, surpassing infectious causes.^3-6,10^ Cardiovascular disease, stroke, chronic respiratory illness, and diabetes remain the principal causes of premature mortality across all regions.^3-6^ These same conditions dominate ICU admissions in both high and low-income settings, illustrating the global epidemiologic transition toward chronic lifestyle-related diseases.^11^ These trends align in the Nepali context as well where infectious causes once dominated but are now being replaced by chronic and lifestyle-related diseases that require complex ICU care.^3,5,11,12^

In Nepal, national mortality estimates indicate that ischemic heart disease, COPD, and stroke are the main causes of death, that mirror the global pattern.^3,5,12^ The predominance of these conditions in our ICU cohort is similar to the findings from other centers in the country,^13,14^ which emphasizes the impact of chronic comorbidities on critical care outcomes.Large multicenter ICU audits, such as the Intensive Care over Nations (ICON) study and other international analyses, report that infection, sepsis, and multiorgan failure remain the most common final pathways of death in critically ill patients.^14-16^ Our findings are consistent with international ICU audits such as ICON and the global JAMA studies, where infection and sepsis are frequent causes of death.^15,16^

Sex-based differences in critical care outcomes have been increasingly recognized globally, with variations in disease presentation, physiological responses, and healthcare access patterns.^17^ However, such differences remain poorly characterized in the Nepali context, where cultural and socioeconomic factors may uniquely influence healthcare seeking behavior and disease patterns between men and women.

This sex-based difference is likely multifactorial which includes chronic exposure to biomass-fuel smoke and delayed access to healthcare among women in rural Nepal thereby increasing respiratory-disease risk.^11,18^ Prior research has also observed gender differences in cardiovascular physiology and ICU outcomes, with women often presenting later in the disease course.^19^ Previous Nepali studies, such as those by Paudel etal. and Koirala et al., have reported similar gender variations, with women presenting later and in more severe condition.^13,14^ The GBD 2021 update emphasized that hyperglycemia, hypertension, and obesity are dominant metabolic risks contributing to mortality in South Asia.^4,5^ This relationship indicates the requirement for integrated management protocols that link chronic disease clinics with hospital critical care systems.

Despite this, Nepal continues to have a relatively low ICU bed-to-population ratio compared to other developing nations, and access to critical care remains limited outside major cities.^12-14^ Most ICUs are located within tertiary hospitals, and referral delays from peripheral centers often worsen outcomes.^3,5,11,20^ Despite these changing disease patterns, intensive care infrastructure in low- and middle-income countries remains inadequate, resulting in poorer survival outcomes.^20-22^ Furthermore, the organization of critical care services in Nepal is still evolving, with variability in case-mix, staffing ratios, and monitoring facilities.^21^ The limited national ICU registries or standardized reporting frameworks has limited systematic analysis of mortality patterns. Understanding how demographic and clinical factors interact to influence ICU deaths can help guide resource allocation and training priorities.^12,13,20^ Understanding local mortality patterns and associated comorbidities is essential for aligning critical care priorities with national disease burdens highlighted by GBD studies.^3-6^

In resource-limited countries like ours, systemic challenges such as shortages of trained staff, lack of monitoring, delayed referral and delayed interventions exacerbate outcomes.^14,20^ Interventions such as early warning systems, rigorous infection control, and point-of-care testing could be implemented in Nepalese ICUs to improve survival.^14,16^ Additionally, continuous education programs and simulation-based training for ICU staff would help strengthen response capacity in resource-limited environments.^14,16,20^

Local autopsy data from Palpa reveal unnatural causes as a significant burden, with road traffic accidents (RTA) foremost cause at 28.8%, followed by hanging (23.4%) and burns (17.9%).^23^ Similarly, a 5-year review at Lumbini Medical College showed pesticides (e.g., organophosphates) as the primary agent in 57.7% of poisoning cases, predominantly self-harm among young females (median age 26.5 years) the underlying cause being family conflicts.^24^ RTA admissions to emergency department of Lumbini Medical College further highlight two-wheelers (46.4%) and passengers (38.4%) as high-risk groups, with a mean arrival time of 3:44 hours post-accident.^25^ This contrast points to a significant limitation: our analysis excludes ICU deaths from unnatural causes (e.g., RTA, poisoning, selfharm), as such cases require police notification and post-mortem examination, precluding death certificate issuance and inclusion in hospital mortality records.

There is an urgent need for public-health approaches that combine preventive measures with hospital care. Reducing household air pollution, promoting healthy lifestyles, and strengthening primary care could decrease cardio-respiratory disease burden.^12,18^ Regular follow-up and better control of blood glucose and hypertension can help reduce admissions to the ICU among high-risk groups.^4,5,15^ Strengthening hospital-based data collection systems and linking them with national health databases could enhance the accuracy of critical care research. Integrating ICU data would inform national policy by providing real-time information on critical care usage and revealing regional differences, which would ultimately lead to better patient care.

The sample size of this study is limited and may have limited the capability to detect statistically significant differences. Additionally, being a cross-sectional observational study, it cannot establish causality. The influence of treatment protocols, medication use prior to admission, or disease etiology was not assessed, which may confound some findings. Exclusion of medico-legal deaths may underestimate true ICU burden, particularly from poisoning and trauma-common in young females and two-wheeler users in Western Nepal.^23,24^

Future research in Nepal should focus on multicenter and prospective designs incorporating cause-specific mortality, and severity scoring systems. Such data would enable national benchmarking and assist policymakers in prioritizing investment in critical care infrastructure and human resources.^13,14,19^ Establishing a national ICU registry, integrating chronic disease clinics with critical care, and fostering collaborations between district hospitals and tertiary centers (e.g., for medico-legal expertise) would enhance surveillance and resource allocation in Nepal’s resource-limited settings.

## CONCLUSION

This study shows sex and comorbidity-related differences in ICU mortality due to natural cause. There was a clustering of mortality within a four-month period from August to November.

## Data Availability

The data are available from the corresponding author upon reasonable request.
